# The ketogenic diet in children 3 years of age or younger: a 10-year single-center experience

**DOI:** 10.1038/s41598-019-45147-6

**Published:** 2019-06-19

**Authors:** Se Hee Kim, Alexandra Shaw, Robyn Blackford, Wesley Lowman, Linda C. Laux, John J. Millichap, Douglas R. Nordli

**Affiliations:** 1 Epilepsy Center, Division of Neurology, Ann & Robert H. Lurie Children’s Hospital of Chicago; Departments of Pediatrics and Neurology, Northwestern University Feinberg School of Medicine, Chicago, Illinois 60611 USA; 2 Department of Clinical Nutrition, Ann & Robert H. Lurie Children’s Hospital of Chicago, Chicago, Illinois 60611 USA; 3 Department of Pediatrics, Division of Neurology, Epilepsy Research Institute, Severance Children’s Hospital, Yonsei University College of Medicine, Seoul, South Korea; 4 Section of Child Neurology, Department of Pediatrics, University of Chicago Pritzker School of Medicine, Chicago, IL USA

**Keywords:** Paediatric research, Epilepsy

## Abstract

The ketogenic diet (KD) is an effective treatment option for intractable epilepsy. Here, we reviewed the last 10 years of our experience with the KD and characterized its use in patients under 3 years of age. Medical records of all patients under the age of 3 years who were treated with the ketogenic diet from April 2004 to June 2014 were retrospectively reviewed. One hundred and nine patients with drug-resistant epilepsy were included. The mean age at the initiation of the KD was 1.4 ± 0.8 years old. The youngest patient was 3 weeks old. After 3 months, 39% (42/109) of patients responded to the KD and experienced more than 50% seizure reduction. Of those 42 patients, 20 (18%) achieved complete seizure control. Patients with a genetic etiology showed a better response to the KD in seizure reduction than the other patients (p = 0.03). Age at initiation of the KD was not related to eventual seizure outcome (p = 0.6). The KD continues to be an effective, safe, and well tolerated treatment option for infants with intractable epilepsy.

## Introduction

The ketogenic diet (KD) is a high fat, low carbohydrate, and protein restricted diet that is rigorously medically supervised and widely recognized as an effective treatment option for intractable epilepsy. It’s use in the United States dates to the 1920s, when Woodyatt and Wilder independently described the production of beta-hydroxybutyric acid or ketonemia via dietary manipulation other than fasting^1,2^.

In a randomized controlled trial analyzing 103 children of varying ages, seizure types, and epilepsy syndromes, 38% of children treated with the KD after 3 months had greater than 50% seizure reduction compared with 6% of controls using heterogeneous anticonvulsant therapies. 7% in the diet group had greater than 90% seizure reduction compared with none of the controls^3^. Overall, while the KD’s use as a therapy for infantile spasms (IS) is perhaps the best known and investigated^4–7^, its effectiveness, safety, and tolerability for infantile onset intractable epilepsy regardless of seizure type has started to be elaborated^7–9^. A recent survey of health care professionals on the use of the ketogenic diet updated the long held notion that it was a therapy of “last resort.” Within sub-types of infantile onset epilepsy, it was considered the first or second treatment for glucose transporter protein 1 deficiency (GLUT1) (86%) and third or fourth for Dravet (63%) and West (71%) syndromes^10^. Despite this, various aspects of implementation and utility of the KD in infants remain less completely understood, including: predictors of favorable response, the combined use of extracted breast milk, potential adverse events, reasons for early withdrawal, as well as the diet’s efficacy in management of specific epilepsy syndromes and etiologies. How advances in medical genomics and the burgeoning identification of causative gene mutations in epilepsy might eventually apply to the ketogenic diet is of considerable interest.

The ketogenic diet has been in continued use at Children’s Memorial Hospital (renamed Ann & Robert H. Lurie Children’s Hospital of Chicago) since 1963^11^. In this study, we retrospectively reviewed the last 10 years of our experience with the KD and characterized its use in patients under 3 years of age. Our goals were to (1) identify predictors of favorable outcome, (2) investigate reasons for early withdrawal with relation to age, diet formulation, or adverse events, and (3) to evaluate efficacy with specific regard to epilepsy syndrome or etiology, utilizing genetic diagnoses if available.

## Methods

Information regarding patient demographics, seizure types, seizure frequency, epilepsy syndrome, etiology, MRI findings and electroencephalogram findings was retrospectively abstracted from the medical records of all patients under the age of 3 years who were treated with the ketogenic diet from April 2004 to June 2014 at the Lurie Children’s (formerly Children’s Memorial Hospital). All patients had medically intractable epilepsy, defined as persistent seizures despite the use of two or more appropriate anticonvulsants at therapeutic doses.

**Figure 1:**
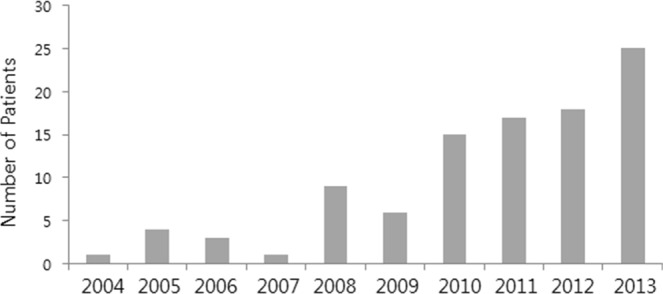
Chart showing the use of the KD in patients under 3 years old in each year (From April 2004 to June 2014). (N. of patients in each year).

Early withdrawal and early discontinuation were defined as cessation of the KD before 3 months of treatment had elapsed. In our analysis, we considered early withdrawal, early discontinuation, and non-adherence in aggregate. Patients were then categorized into two groups: (1) an early withdrawal group and (2) an adherent group (Fig. 1). To determine the reasons for early withdrawal, three clinical features were reviewed: very short-term efficacy of the KD, adverse events, and the implementation and practice of the KD. The number of patients who withdrew from the diet early because of ineffectiveness was calculated.

Further, the patients who completed 3 months on the KD were categorized into four groups based on the percentage of seizure reduction at 3 months: (1) complete seizure control, (2) >90% improvement, (3) 50–90% improvement, and (4) <50% improvement. Responders were defined as those who experienced >50% seizure reduction. Non-responders were defined as those who had <50% seizure reduction. Seizure frequency was reported by the parents as well as by EEGs when available. We defined adverse events broadly, including both new clinical symptomatology and lab abnormalities that started while using the KD. Events that prompted early withdrawal were also specified. To evaluate the practice details of KD implementation and maintenance, we assessed the ratio (grams of fat to grams of carbohydrate + protein) used, the method of food consumption, and the type of food that was consumed. Finally, in order to elucidate any age-dependent outcomes, patients were further categorized into three groups based on their age at the KD initiation and evaluated as follows: (1) patients <1 year old, (2) 1–2 years old, and (3) >2 years old. The study was approved by the Lurie Children’s Hospital Institutional Review Board. All research was performed in accordance with relevant guidelines/regulations and a waiver of informed consent was granted for this retrospective study.

### The KD protocol

Before initiation of the KD, a multidisciplinary evaluation was performed by two registered dietitians, advanced practice nurses, and by a licensed clinical social worker. During the visit, a review of the medical history was obtained, diet protocols and lifestyle implications were discussed, and a neurologic examination was completed. After this preliminary visit, one of the following combinations of food consumption mechanism and food type were selected: oral liquid, tube-fed liquid, oral solids, or a combination of oral liquid and solid foods. Liquid foods included: individually designed meals with oils as well as commercial formulas including KetoCal® (Nutricia Inc., Gaithersburg, MD, U.S.A.) and Ross Carbohydrate Free® Soy Formula Base With Iron (Abbott Nutrition, Columbus, OH, USA). Individual calorie and protein amounts were calculated by our dietitians based on age-dependent standard daily requirements, baseline weight and height, level of activity, and the pre-diet intake evaluation. Supplementation with vitamins and minerals was provided based on individual patients’ needs and food types used. The KD was initiated at a ratio of 1:1 (fat grams: carbohydrate + protein grams) without a fast as an inpatient in the hospital. The keto ratio was increased daily, reaching up to 3:1 on day 3. On day 4, patients were discharged home at the ratio of 3:1 with full calories. Fluids were not restricted. Patients were then evaluated by our multidisciplinary KD team 1 month later and then every 3 months thereafter. Intermittent outpatient adjustments of diet ratio, number of calories, and amount of protein were made to maximize seizure control, minimize side effects, and maintain appropriate growth.

### Statistical analysis

In order to determine the factors that were associated with the early withdrawal of the KD, we compared the clinical variables between the early withdrawal group and the continuing (adherent) group. We used the independent *t*-test or the Mann-Whitney *U* test to analyze continuous variables including the age at KD initiation, age at seizure onset, seizure onset prior to the KD initiation, duration of the KD, and the number of prior antiepileptic drugs. For categorical data, Pearson’s chi-square test for independence of rows and columns was used. *P* values < 0.05 were regarded as statistically significant. Statistical analyses were performed using SPSS version 22 (SPSS Inc., Chicago, IL, USA). Data are expressed as number (%), mean ± standard deviation, or median (interquartile range (IQR)).

## Results

### Baseline characteristics

**Figure 2:**
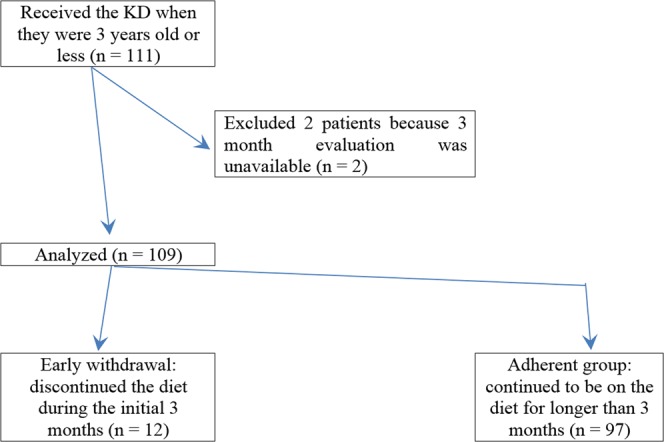
Flow diagram.

One hundred and eleven patients under 3 years of age were started on the KD (Fig. 1). Two patients were excluded from the study: one underwent epilepsy surgery after one month of the treatment with the KD; the other, a patient with early myoclonic encephalopathy (EME), died due to respiratory failure 2 months into treatment with the KD. Ultimately, one hundred and nine patients with drug-resistant epilepsy were included for analysis (Fig. 2). Details of baseline patient characteristics are given in Table 1. The median age at seizure onset was 4 (IQR 1–6) months of age. The median number of previously attempted and/or concomitantly used medications at the time of the KD initiation was 4 (IQR 3–5). Approximately half (56%) of the patients had West syndrome. The EEG was abnormal in all patients at initiation of the diet.

**Table 1:** Patient characteristics (n = 109).

		Values
Males/Females, n		50/59
Age at the seizure onset, median (IQR), y		0.3 (0.1–0.5)
Age at the initiation of the KD, mean ± SD, y		1.4 ± 0.8
Duration of the KD, median (IQR), y		1.1 (0.5–2.2)
N of prior anticonvulsants, median (IQR)		4 (3–5)
Epilepsy syndrome, n(%)	West syndrome	61 (56)
	Dravet syndrome	9 (8)
	Localization related epilepsy	7 (6)
	LIEE	5 (5)
	EIEE/EME	3 (3)
	Doose syndrome	2 (2)
	Myoclonic epilepsy	2 (2)
	MMPI	1 (1)
	Unknown	19 (18)
Etiology, n(%)	Structural	24 (22)
	Perinatal events (HIE or IVH)	20 (18)
	Genetic (chromosomal abnormalities or single gene defect)	14 (13)
	Channelopathy	13 (12)
	Metabolic disorder	8 (7)
	Leukodystrophy	4 (4)
	Trauma	3 (3)
	Unknown	23 (21)
EEG findings, n(%)	Diffuse slowing with Mf spikes + electrodecrements	47 (43)
	Diffuse slowing with Mf spikes	38 (35)
	Focal slowing and spikes + electrodecrements	14 (13)
	Focal slowing and spikes	9 (8)
	Generalized spike wave discharges with no slowing	1 (1)
Outcome, n(%)	Seizure free	20 (18)
	Reduction >90%	3 (3)
	Reduction 50–90%	19 (17)
	Reduction <50%	55 (50)
	Withdrawal during the initial 3 months	12 (11)

KD, ketogenic diet; N, number; IQR, interquartile range; SD, standard deviation; LIEE, late infantile epileptic encephalopathy; EIEE, early infantile epileptic encephalopathy; EME, early myoclonic encephalopathy; MMFSI, malignant migrating focal seizures in infancy; HIE, hypoxic ischemic encephalopathy; IVH, intravenous hemorrhage; EEG, electroencephalogram.

The mean age at the initiation of the KD was 1.4 ± 0.8 years old. The youngest patient was 3 weeks old. Among 105 patients for whom information on the KD ratio was available, 20 were on a 3:1 ratio, 13 were on 3.5:1 and 59 were on 4:1. Overall, 12 were on a ratio higher than 4:1. The maximum ratio used was 4.75:1. Every child reached a fat to non-fat ratio of 3:1 or higher except one who was on a ratio of 2.75:1. The form of the food that was used included solid foods (33%, 35/104), liquid foods (32%, 34/104) and a combination of both (33%, 35/104). **[**Among 69 patients who received a liquid form of the KD, 96% (66/69) utilized the ketogenic formula while 4% (3/69) were put on an individually designed liquid. Eight patients used expressed breast milk. The median duration of the KD was 1.1 (IQR 0.5–2.2) years. All but one patient had positive urine ketosis at some point during the diet. In the one outlier, the level of serum *B*-hydroxybutyrate was 1.32 mmol/L and seizures did not respond (seizure reduction <50%) to the 3 months of the diet therapy.

### Efficacy

After 3 months, 39% (42/109) of patients responded to the KD and experienced more than 50% seizure reduction. Of those 42 patients, 20 (18%) achieved complete seizure control and an additional 3 (3%) experienced >90% seizure reduction (Table 1). There was no significant difference between age at the seizure onset (p = 0.2) or age at initiation of the KD (p = 0.5) between responders and non-responders. The number of previously or currently used anticonvulsants was lower among responders (p = 0.02).

Possibly due to the small numbers of patients, no specific etiology was significantly related to a positive response to the KD (Table 2). Interestingly, however, patients with a confirmed genetic mutation or a chromosomal abnormality showed a better response to the KD in seizure reduction than the other patients (p = 0.03). Genetic abnormalities were identified in 29 patients and included mutations of the following genes: *BRAF*, *CDG 1p*, *CDKL5*, *SCN1A*, *SCN2A*, *SCN8A*, *KCNQ2*, *DCX*, *EEF1A2*, *GFAP*, *GRIN2A*, *MTO*, *POLG*, *RARS2*, *TSC2*, *SLC35A2* and *SLC6A8*. Trisomy 21 was found in 5 patients. Microdeletions of chromosome 1, 6 and 16 were found in 5 patients. The KD was similarly effective in patients with diverse early onset epilepsies and in patients with West syndrome.

**Table 2:** Differences between responders (seizure reduction >50%) and the non-responders (seizure reduction <50%) on the ketogenic diet.

		Responder	Non-responder	p-value
n		42	67	
Age at the seizure onset, median (IQR), y		3 (2–6)	3 (1–3)	0.2
Age at the initiation of the KD, mean ± SD, y		1 ± 0.8	1 ± 0.8	0.5
N of prior anticonvulsants, median (IQR)		3 ± 1.6	4 ± 1.9	0.02
Risk factor	Positive MRI finding	20	34	0.7
	Genetic abnormality	20	19	0.03
	Metabolic abnormality	3	7	0.6
Etiology	Channelopathy	5	7	0.9
	HIE or IVH	9	8	0.2
	Leukodystrophy or Metabolic disorder	4	7	0.9
	MCD or TS	6	17	0.2
Epilepsy syndrome	West syndrome	24	36	0.7
	Non-West syndrome	18	31	
EEG findings, *n* = 108	a. Diffuse slowing with Mf spikes	16	22	0.4
	b. Diffuse slowing with Mf spikes + electrodecrements	18	29	
	c. Focal slowing and spikes	5	4	
	d. Focal slowing and spikes + electrodecrements	3	11	

KD, ketogenic diet; IQR, interquartile range; SD, standard deviation; N, number; MRI, magnetic resonance image; HIE, hypoxic ischemic encephalopathy; IVH, intravenous hemorrhage; MCD, malformation of cortical development; TS, tuberous sclerosis; Mf, multifocal.

In general, there was no single EEG abnormality that was independently related to a favorable response to the KD. Among patients with focal slowing and spikes on the baseline EEG, the presence of electrodecrements was suggestive of a poor response, but failed to show a significant difference (p = 0.09).

### Early withdrawal and associated factors

Only 12 of 109 patients (11%) withdrew from the diet before 3 months. The most common cause for withdrawal was parental unhappiness with the diet’s rigidity (7/12). Only five patients stopped early either because of perceived ineffectiveness (3/12) or because of an adverse event (2/12) (Table 3). Young patients who were given ketogenic formula with or without solids were more likely to continue the diet (p = 0.003). A high KD ratio (≥4:1), the use of feeding tube or the use of expressed breast milk was not related to early KD withdrawal. Young patients who were given ketogenic formula were more likely to continue the diet (p = 0.003). A high KD ratio (≥4:1), the use of feeding tube or the use of expressed breast milk was not related to early KD withdrawal.

**Table 3:** Reasons for early withdrawal (n = 109).

Reason	N (%)
Parental unhappiness	7 (6)
Ineffectiveness	3 (3)
Adverse event	2 (2)

Young patients who were given ketogenic formula were more likely to continue the diet (p = 0.003). A high KD ratio (≥4:1), the use of feeding tube or the use of expressed breast milk was not related to early KD withdrawal.

The two adverse events that caused early withdrawal were behavioral food refusal and persistent acidosis in the setting of poor food intake. No one specific adverse event occurred more frequently among the early withdrawals. Constipation was more likely to be reported in the adherent group who were on the diet for a longer period of time (p = 0.009). Further details of the adverse events are provided in Table 4.

**Table 4:** Differences between the patients who continued to be on the KD and the patients who withdrew early before they completed the 3 months of the KD therapy.

		Total	Adherent group	Early withdrawal group	p-value
N		109	97	12	
Male/Female		50/59	44/53	6/6	0.8
Age at the initiation of the KD, mean ± SD, *y*		1.4 ± 0.8	1.4 ± 0.8	1.4 ± 0.8	1
N of prior anticonvulsants, *n* = 95		4 ± 1.9	4 ± 1.9	4 ± 1.8	0.7
Improved cognition, *n* = 95		47	47	0	0.007
High KD ratio ≥ 4:1		71	67	4	0.07
Food type, *n* = 104	Solid	35	28	7	0.1
	Liquid	34	32	2	
	Solid and liquid	35	32	3	
Feeding tube, *n* = 105	49	46	3	0.1	
Use of a solid food		70	60	10	0.2
Ketogenic formula		66	63	3	0.003
Expressed breast milk	8	7	1	0.9	
AE or abnormal lab result	69	65	4	0.01	
	Constipation	35	35	0	0.009
	Decreased HCO_3_^−^ level*	36	34	2	0.2
	Vomiting, reflux	22	11	11	0.7
	Low free carnitine level^#^	9	9	0	0.3
	Feeding difficulty	6	5	1	0.7
	Kidney stone	3	3	0	0.5
	Transient hypoglycemia	2	2	0	0.6
	Others	7	7	0	0.3
AE which required a management	63	59	4	0.1	

KD, ketogenic diet; SD, standard deviation; N, number; AE, adverse event; KD. *Decreased HCO3- level (<18 mEq/L). ^#^Low free carnitine level (<27 µmol/L).

### The effects of age

Age at initiation of the KD was not related to eventual seizure outcome (p = 0.6). Likewise, age was not related to early withdrawal from the KD (p = 0.2). Interestingly, however, the age group most likely to withdraw from the KD early was group 2 (patients between 1 and 2 years old) (7/12, 58%). This group also showed a wider variety of food types used during the KD compared to the other two groups whose food options were more selective. As would be expected, formula was used more in younger patients (<1 year old), In older patients (>2 years old), solid foods were more predominantly used. In patients between 1 and 2 years old, no single predominant food type was found (p < 0.0001). Details are given in Table 5. The overall occurrence of an adverse event was not related to age (p = 0.6). Patients who started the KD earlier (before 2 years old), however, were more likely to report constipation (p = 0.005). Kidney stones were observed only in patients younger than 1, but the difference according to age was not statistically significant (p = 0.07). Patients who discontinued the KD early because of an adverse event were 1.9 and 2.6 years old at the time of the KD initiation.

**Table 5:** Age-related differences.

		Total	<1year old	1–2 year old	>2 year old	P-value
N		109	44	38	27	
Female		59	23	23	13	0.6
West syndrome		60	28	24	8	0.0009
Outcome at 3 months	Seizure free	20	10	6	4	0.6
	>90% reduction	3	2	0	1	
	50–90% reduction	19	8	4	7	
	<50% reduction	55	21	21	13	
Discontinuation before 3mo	12	3	7	2	0.2	
Improved cognition per parent, *n* = 95	47	23	14	10	0.4	
High KD ratio ≥ 4:1		71	37	18	16	0.001
Tube-fed		49	23	17	9	0.4
Expressed breast milk		8	8	0	0	0.002
Food type, *n* = 104	Solid	35	4	17	14	<0.0001
	Liquid	34	16	11	7	
	Solid and liquid	35	22	10	3	
Ketogenic formula, *n* = 104	66	37	20	9	<0.0001	
AE or abnormal lab results	69	30	25	14	0.6	
	Constipation	35	22	9	4	0.005
Decreased HCO_3_^−^ level	36	18	9	9	0.2	
	Vomiting, reflux	12	5	5	2	0.8
Low free carnitine level	9	4	5	0	0.07	
	Feeding difficulty	6	0	3	3	0.08
	Kidney stone	3	3	0	0	0.07
Transient hypoglycemia	2	1	1	0	0.7	
	Others	7	5	2	0	0.2
AE which required a management	63	29	23	11	0.01	

AE, adverse event; KD, ketogenic diet. *Decreased HCO3- level (<18 mEq/L). Low free carnitine level (<27 µmol/L).

## Discussion

Within a span of 10 years at our center, more than 100 patients were initiated on the KD before they turned 3 years old. Overall, 20% of patients became seizure free and many (39%) experienced a significant seizure reduction (>50%) after the initial 3-months of the KD therapy. This trend is similar to what other studies have reported for older children, in which 27–38% of children have a >50% seizure reduction after 3 months^3,12^. The results were also comparable to the rates of seizure reduction reported for the infants with West syndrome, which ranged from 39% to 67%^4,6,13^.

Our previous study was the first to investigate the role of the KD in infants with epilepsies other than West syndrome/infantile spasms^7^. Fifteen years ago, we had only a limited ability to assess the efficacy of the KD with etiologic specificity. Since then, there have been smaller studies and case series or case reports looking at the diet’s role in individual epilepsy syndromes or diseases that could include an infantile presentation, such as: GLUT-1 deficiency syndrome, pyruvate dehydrogenase deficiency (PDHD), severe myoclonic epilepsy of infancy (Dravet syndrome), Rett syndrome, and selected mitochondrial disorders^14–17^. None of these studies, however, focused exclusively on early introduction of the KD during the time period of 3 years of age and younger. It is would be of great benefit to determine which epilepsy syndromes, beyond West syndrome, and which etiologies are more responsive to the KD, so as to promote early referral and initiation of treatment. In the present study, there was no significant difference between the effectiveness of the KD for patients with West syndrome and other epilepsy syndromes, which suggests that the KD should be considered early in all infants with refractory epilepsy once contraindications are ruled out.

Moreover, in our experience, a confirmed genetic abnormality was predictive of a good response to the KD. Nearly half of the patients with a confirmed genetic abnormality enjoyed a reduction in seizure frequency of >50%. A similar finding was described in a retrospective chart review of 64 consecutive patients with refractory epilepsy, children and adults, who were started on the KD at a tertiary epilepsy center^18^. This finding implies that the KD could also be considered by providers earlier, perhaps even before a patient fails two previous anticonvulsant medications, if he or she receives a positive genetic diagnosis. Furthermore, early diet initiation not only capitalizes on age-related ease of use and tolerability, but could also appeal to parents concerned about potential anticonvulsant side effects, a finding shared in other studies^8,19^.

In our experience with 109 children under 3 years of age, the KD was safely implemented and tolerated for the initial 3 months. Only about 10% of the children withdrew early. Among these, we found no critical adverse event related to use of the KD. The rate of early withdrawal was comparable to those reported in older children, which ranged from 12% to 16^3,12,20^. A much higher number of early withdrawals have been reported in a study with adults. Implementation and maintenance of the KD was easier in young children whose diet habits are still flexible, a finding supported by previous studies with smaller sample sizes which found similarly low rates of early withdrawal in of children in this age group (<2 years old)^4,5,7,13^.

Considering that this study was performed at a tertiary pediatric epilepsy center with a specialized KD team, it was surprising to find that parental dissatisfaction with the diet was the most commonly reported cause for early withdrawal. This finding emphasizes the extensive amount of multidisciplinary support that parents need while using the diet, especially during the complicated period of solid food introduction.

Adverse events were a rare cause of early withdrawal in children (2%), although many children experienced at least one during their 3 months use of the KD (63%, 69/109). This finding is in partial agreement with previous reports which described that approximately 30% of the children experience an adverse event, but only 3–5% of children discontinue the diet due to adverse events^4,5^. We suspect that the reported rate of adverse events was higher in our study because our pre-determined threshold for adverse event reporting was intentionally set low. For instance, symptoms as mild as feeding difficulty or self-limited, small emesis were included as adverse events, which were in turn related to the fastidiousness of parental report or clinical judgement of the involved health care professional. Additionally, laboratory abnormalities such as decreased bicarbonate or glucose levels which were described in other studies but not reported as adverse events were counted as such in ours. We believe that the comprehensiveness of our reporting will be helpful in guiding physicians and caregivers when they plan to start the diet in infants and toddlers.

There was a significant relationship between early withdrawal from the diet and the food type that was used. While a few case-series and short studies have suggested improved tolerability of the liquid-based KD^4,21–23^, its use has never been studied exclusively in infants, the group that not only makes most frequent use of this formulation, but who may also benefit most from it. Our study demonstrates that a liquid-based KD with ketogenic formula can be used safely and effectively in infants and that it may even enhance patient and parental maintenance of the diet. We also report here on eight children who successfully completed the diet with a combination of formula and expressed breast milk. The use of breast milk was neither related to early withdrawal, nor to poor outcome in terms of seizure control. This finding suggests that consideration of ketogenic diet therapy should not reflexively prompt early weaning from breast milk.

We did not find any age-related difference in regard to the outcome or the practice of the KD therapy in young children. Some studies have suggested a young age at the time of the KD to be a favorable factor while others did^7,20,24^. In our study, age was not related to the seizure outcome but to the methods of the KD and to the adverse events. Younger ones (<1 year old) were more likely to be fed with the liquid KD food while older ones (>2 years old) were fed with the solid foods. Younger ones were more likely to report constipation and low carnitine levels compared to the older ones. Additionally, although we failed to see a statistically significant difference, we also saw an age-related trend in relation to early withdrawal. Interestingly, it was not the older children (>2 years old) or the younger ones (<1 year old), but the children who started the KD between 1 and 2 years old, who tended to withdraw early from the diet more frequently than the other two groups (p = 0.15). We hypothesize that applying the KD may be more challenging to children when they are being introduced to the solid foods for the first time. Restricting the diet or measuring the amount can be more difficult for the parents during this period. Our data regarding the selected food options supports this finding in which a bigger variation was reported among children aged between 1 and 2 years old in comparison to the other two age groups in which one type of foods (solid vs. liquid) were predominantly used (p = 0.001).

Patient growth data were not included. Some studies have reported osteopenia, growth arrest and weight loss in children on the KD^25,26^. It would be beneficial to learn how to address these issues in a more focused manner in the infant period, however, they may be more properly addressed in a long-term evaluation in collaboration with an endocrinologist. At present, the role of ketosis during the diet remains unknown. In our study, potential relationships between ketosis and seizure frequency or withdrawal from the KD was not evaluated. Technical limitation: urine ketones positive for most of the patients. Additionally, because of the retrospective nature of the study, it was difficult to identify the temporal relationship between urine ketones and seizure reduction. A recent study demonstrated the inconsistent relationship between ketosis and good seizure control in infants^12,13^. The fidelity of this relationship could be addressed in a future study. Lastly, the context in which this study was completed - a comprehensive epilepsy center with two dietitians working exclusively on the KD – may have lowered the rate of early withdrawal. On the other hand, because of a high volume of referrals for intractable epilepsy, we may have selectively included children with the most severe forms of epilepsy. Despite these limitations, the study has clinical significance demonstrating a safe and variable use of KD in young children. These findings will facilitate the safe use of the diet therapy in this population.

## Conclusion

The KD continues to be an effective, safe, and well tolerated treatment option for infants with intractable epilepsy. Tolerability may be enhanced by using a liquid-based diet in this age group, the role of expressed breast milk in which should be further elaborated. The availability of new diagnostic studies such as genetic testing could promote early and effective use of the KD by identifying patients who might favorably respond to the KD. Future studies with larger patient sample sizes require multi-center collaboration and would further refine our understanding of which genetic abnormalities and epilepsy syndromes respond best to the ketogenic diet.

## Data Availability

The datasets generated during and/or analysed during the current study are available from the corresponding author upon reasonable request.

## References

[1] Woodyatt RT (1921). Objects and method of diet adjustment in diabetes. Archives of Internal Medicine.

[2] RM W (1921). The effects of ketonemia on the course of epilepsy. Mayo Clin Bull.

[3] Neal EG (2009). A randomized trial of classical and medium-chain triglyceride ketogenic diets in the treatment of childhood epilepsy. Epilepsia.

[4] Eun SH, Kang HC, Kim DW, Kim HD (2006). Ketogenic diet for treatment of infantile spasms. Brain & Development.

[5] Hong AM, Turner Z, Hamdy RF, Kossoff EH (2010). Infantile spasms treated with the ketogenic diet: prospective single-center experience in 104 consecutive infants. Epilepsia.

[6] Kossoff EH, Pyzik PL, McGrogan JR, Vining EPG, Freeman JM (2002). Efficacy of the ketogenic diet for infantile spasms. Pediatrics.

[7] Nordli DR (2001). Experience with the ketogenic diet in infants. Pediatrics.

[8] Dressler A (2015). The ketogenic diet in infants–Advantages of early use. Epilepsy Research.

[9] Kossoff EH (2018). Optimal clinical management of children receiving dietary therapies for epilepsy: Updated recommendations of the International Ketogenic Diet Study Group. Epilepsia Open.

[10] Jung DE, Joshi SM, Berg AT (2015). How do you keto? Survey of North American pediatric ketogenic diet centers. Journal of Child Neurology.

[11] Millichap JG, Jones JD, Rudis BP (1964). Mechanism of anticonvulsant action of ketogenic diet. Studies in animals with experimental seizures and in children with petit mal epilepsy. American Journal of Diseases of Children.

[12] Neal EG (2008). The ketogenic diet for the treatment of childhood epilepsy: a randomised controlled trial. Lancet Neurology.

[13] Numis AL, Yellen MB, Chu-Shore CJ, Pfeifer HH, Thiele EA (2011). The relationship of ketosis and growth to the efficacy of the ketogenic diet in infantile spasms. Epilepsy Research.

[14] Gano LB, Patel M, Rho JM (2014). Ketogenic diets, mitochondria, and neurological diseases. Journal of Lipid Research.

[15] Liebhaber GM, Riemann E, Baumeister FAM (2003). Ketogenic diet in Rett syndrome. Journal of Child Neurology.

[16] Ramm-Pettersen A (2013). Good outcome in patients with early dietary treatment of GLUT-1 deficiency syndrome: results from a retrospective Norwegian study. Developmental Medicine and Child Neurology.

[17] Sofou K (2017). Ketogenic diet in pyruvate dehydrogenase complex deficiency: short- and long-term outcomes. Journal of Inherited Metabolic Disease.

[18] Thammongkol S (2012). Efficacy of the ketogenic diet: which epilepsies respond?. Epilepsia.

[19] Rubenstein JE (2005). Experience in the use of the ketogenic diet as early therapy. Journal of Child Neurology.

[20] Vining EP (1998). A multicenter study of the efficacy of the ketogenic diet. Archives of Neurology.

[21] Kang HC, Kim HD, Kim DW (2006). Short-term trial of a liquid ketogenic milk to infants with West syndrome. Brain & Development.

[22] Kossoff EH, Dorward JL, Turner Z, Pyzik PL (2011). Prospective study of the modified atkins diet in combination with a ketogenic liquid supplement during the initial month. Journal of Child Neurology.

[23] Kossoff EH, McGrogan JR, Freeman JM (2004). Benefits of an all-liquid ketogenic diet. Epilepsia.

[24] Suo C (2013). Efficacy and safety of the ketogenic diet in Chinese children. Seizure.

[25] Groleau V, Schall JI, Stallings VA, Bergqvist CA (2014). Long-term impact of the ketogenic diet on growth and resting energy expenditure in children with intractable epilepsy. Dev Med Child Neurol.

[26] Kang HC, Chung DE, Kim DW, Kim HD (2004). Early- and late-onset complications of the ketogenic diet for intractable epilepsy. Epilepsia.

